# A study on out-of-field leakage of an accelerator-based neutron beam for boron neutron capture therapy

**DOI:** 10.3389/fonc.2023.1284405

**Published:** 2024-02-09

**Authors:** Zhen-Fan You, Chun-Kai Huang, Yen-Wan Hsueh Liu

**Affiliations:** ^1^ Medical Device Technology Development Department, Heron Neutron Medical Corporation, Zhubei, Taiwan; ^2^ Radiation Therapy Technology Development Department, Heron Neutron Medical Corporation, Zhubei, Taiwan; ^3^ Research and Development Center, Heron Neutron Medical Corporation, Zhubei, Taiwan

**Keywords:** boron neutron capture therapy, whole-body dose, out-of-field leakage, neutron beam design, beam profile

## Abstract

More and more accelerator-based boron neutron capture therapy (AB-BNCT) facilities are under the construction or commissioning stage, and the neutron beam characteristic measurements at each facility will start soon. In addition to the in-field neutron beam properties, the leakage of neutron beam is also important, which is related to the side effects of the patient. In the Virtual Technical Meeting on Advances in Boron Neutron Capture Therapy held by International Atomic Energy Agency (IAEA) in July 2020, the issue of the out-of-field leakage in BNCT was addressed. Heron Neutron Medical Corporation has been working on the beam design for China Medical University Hsinchu Hospital AB-BNCT research center. To evaluate the out-of-field leakage, both beam profile analysis and whole-body dose calculation are performed. An Oak Ridge National Laboratory (ORNL) Medical Internal Radiation Dose (MIRD) mathematical phantom is used to calculate the whole-body dose. For the estimated irradiation time which is set to be the time required for 80% of tumor dose to reach 20 Gy-w, the relative biological effectiveness weighted dose of abdomen region is less than 40 mGy-w and the whole-body dose is 104 mSv. The beam profile calculational result shows that the neutron ambient dose equivalent at 15 cm from the field edge is 11 mSv/Gy-w and drops to 5 mSv/Gy-w at 26 cm from the field edge. The gamma ray ambient dose equivalent is less than 1 mSv/Gy-w starting from 10 cm from the field edge. Although the neutron out-of-field leakage of the beam design is higher than that of the initially proposed guideline by IAEA in 2020, the whole-body dose, however, is reasonably low. Both the whole-body dose evaluation and the beam profile analysis are useful in the beam design consideration. The whole-body dose calculation together with the beam profile analysis can also be helpful in reaching an acceptable recommendation for the out-of-field leakage for BNCT neutron beam, a job wished to be accomplished in the near future as proposed in the 2023 IAEA’s report on Advances in Boron Neutron Capture Therapy.

## 1 Introduction

The recommendation for epithermal neutron beam for boron neutron capture therapy (BNCT) has been focused on the beam characteristics at the beam port, such as the in-air beam intensity and quality, and the in-phantom figure of merits (1). In the Virtual Technical Meeting on Advances in Boron Neutron Capture Therapy held by IAEA on 27 to 31 July 2020, the issue of the out-of-field leakage in boron neutron capture therapy was addressed (2). It was further pointed out in the recent issue of the IAEA report “Advances in Neutron Capture Therapy” (3) that the out-of-field leakage dose, also called “non-target radiation,” is of concern because it causes an unnecessary risk of harm to the patient. It is therefore important to take into consideration the out-of-field leakage in the beam shaping assembly (BSA) design.

Heron Neutron Medical Corporation has been working on the beam design for an accelerator-based BNCT facility. In this study, an ORNL MIRD mathematical male phantom (4, 5) is used to calculate the whole-body dose associated with the BNCT treatment of head-and-neck (H&N) cancer. The beam profile and the ambient dose from the beam edge are also calculated.

## 2 Materials and methods

The modeling problem is a 30-MeV/400-μA proton beam bombarding beryllium target. The beam shaping assembly consists of Fe, FLUENTAL™, and MgF_2_. The collimator material is Bi. Lead is used as neutron reflector and for gamma ray shielding. PE(Li_2_CO_3_) and PE(H_2_BO_3_) are used for neutron shielding. The diameter of neutron beam port is 14 cm. The calculation tool is Monte Carlo N–Particle Transport Code System (MCNP6) (6), and the cross-section library is ENDF B VII.0/VII.1 (7, 8).

### 2.1 Whole-body dose calculation

In this study, an ORNL MIRD mathematical male phantom is used to calculate the whole-body dose associated with the BNCT treatment of H&N cancer. Instead of using radiation weighting factor of International Commission on Radiological Protection (ICRP), the relative biological effectiveness (RBE) factor of BNCT is used to obtain the equivalent dose of individual organ $\;H_{T,\;BNCT}$ through Equation 1.


(1) $$H_{T,\;\;BNCT}=\sum_{R}RBE_{R}\times D_{T,R}\;\;unit:Gy-w$$

where $D_{T,R}$ is the average absorbed dose from radiation R in the tissue or organ T, and RBE_R_ is the RBE factor of radiation R. The RBE of gamma ray and neutron is 1 and 3.2, respectively, as commonly used in BNCT treatment. Then, the tissue weighting factor is used for obtaining the whole-body effective dose *E* through Equation 2.


(2) $$E=\sum_{T}W_{T}\times H_{T,\;\;BNCT\;}\;\;\;\;unit:Sv$$

where $W_{T}$ is the tissue weighting factor.

**Figure 1:**
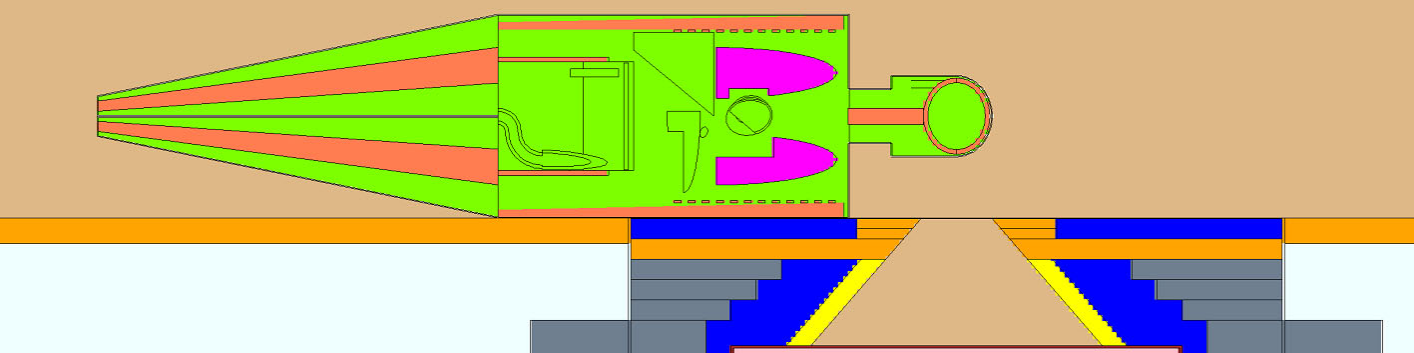
Geometry model of whole-body dose calculation. The yellow color of the beam shaping assembly (BSA) is Bi collimator. The blue color of BSA is Pb. The orange color of the BSA is PE(Li_2_CO_3_). The gray color of the BSA is PE(H_2_BO_3_). The light blue color outside the BSA is concrete.

The incident direction of neutron beam is from the left-hand side of the mathematical phantom with a lying posture, aiming at the center of the brain. The left shoulder is aligned with the surface of the beam exit, as shown in **Figure 1**.

### 2.2 The beam profile and the ambient dose from the field edge

The issue of out-of-field leakage in BNCT was addressed in the Virtual Technical Meeting on Advances in Boron Neutron Capture Therapy on 27 to 31 July 2020, and the preliminarily recommended values for the ambient dose equivalent from neutrons/photons for a distance of 15 cm to 200 cm from the field edge were proposed. The proposed recommended value at that time for the ambient dose equivalent from neutrons is less than 5 mSv/Gy-w for a distance of 15 cm to 200 cm from the field edge, and that for the ambient dose equivalent from photons is less than 5 mSv/Gy-w for a distance to 15 cm to 50 cm from the field edge and is less than 1 mSv/Gy-w for a distance of 50 cm to 200 cm from the field edge. In the 2023 IAEA’s report on Advances in Boron Neutron Capture Therapy (3), it is suggested that, before the reasonable target values for maximum allowed out-of-field leakage dose in BNCT can be specified, the ratio of the leakage dose and the prescribed dose needs to be evaluated for the existing BNCT facilities using the common parameters listed in Table 38 of reference (3).

**Figure 2:**
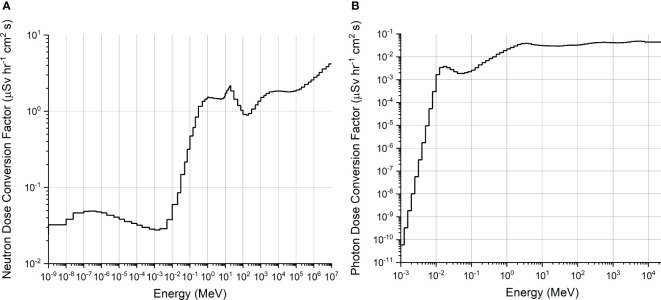
**(A)** Neutron ambient dose conversion factor (µSv∙h^−1^∙cm^2^∙s) (9). **(B)** Photon ambient dose conversion factor (µSv∙h^−1^∙cm^2^∙s) (9).

The indicator for out-of-field leakage to the patient is ambient dose equivalent (Sv/Gy-w) defined as Equation 3.


(3) $$Ambient\;dose\;equivelent=\frac{Ambient\;dose}{maximum\;tumor\;dose}$$

where the maximum tumor dose is the dose to brain tumor in the large phantom positioned so that the distance from the beam exit to dose maximum is 5 cm. The ambient dose conversion factor is based on fits to values for discrete energies as suggested by ICRP74 and as calculated by Pelliccioni et al. with FLUKA (9). **Figure 2** shows neutron ambient dose conversion factor and photon ambient dose conversion factor.

The field edge is the radial distance of the 30% of maximum tumor dose from the beam central axis in the large water phantom defined at the reference depth where the thermal neutron flux is maximum.

The calculation condition is based on a water phantom of 60 cm × 60 cm × 60 cm placed at the neutron beam exit. The boron concentration in blood is 25 ppm. The tumor–to–normal tissue ratio of boron is 3.5. The values of the RBE and the compound biological effectiveness (CBE) suggested in Table 28 of reference (3) are used. The CBE of tumor is 4.0, and the RBE of the nitrogen dose, the hydrogen dose, and the photon dose is 2.9, 2.4, and 1.0, respectively.

## 3 Results

### 3.1 Whole-body dose calculation

**Table 1** shows the RBE-weighted equivalent dose of organs in the whole-body dose calculation. The tissue weighting factor W_T_ is based on ICRP 103 report (10). The irradiation time is set to be the time required for 80% of tumor volume to reach 20 Gy-w, which is estimated to be 18.7 min based on the THORplan (11) treatment planning results for clinical trial of recurrent H&N cancer (12). The summation of BNCT RBE-weighted dose multiplied by tissue weighting factor is analogous to that of the effective dose. The RBE-weighted dose of organs in the abdomen region is less than 40 mGy-w.

**Table 1:** BNCT RBE-weighted equivalent dose of individual organ in the whole-body dose calculation and the corresponding tissue weighting factor.

Organ	$\;H_{T,\;\;BNCT}$ (Gy-w)	W_T_ ^a^
Bone	2.71E-01	0.13^b^
Colon	1.36E-02	0.12
Lung	1.50E-01	0.12
Stomach	3.74E-02	0.12
Breast	– ^c^	0.12
Gonads	6.02E-03	0.08
Bladder	9.74E-03	0.04
Esophagus	7.59E-02	0.04
Liver	2.82E-02	0.04
Thyroid	3.32E-01	0.04
Brain	1.74E+00	0.01
Skin	2.06E-01	0.01
Remainder	5.85E-02	0.13^d^
Total	$\sum \left(H_{T,\;\;BNCT}\cdot W_{T}\right)$	0.104 Sv

a Based on ICRP 103 report;

b Including bone marrow and bone surface;

c Male phantom;

d Including salivary gland.

As shown in **Table 2**, the whole-body dose is 104 mSv, among which 46% comes from the primary beam port of 14-cm diameter, and 69% comes from the virtually extended primary beam port of 22-cm diameter. The neutrons contribute 85% of the total whole-body dose at the beam exit. For the dose contributed from outside the 14-cm diameter beam port, 77% is neutron dose, and half of them are from the ring region between radius of 7 cm and 11 cm.

**Table 2:** Contribution of non-primary beam to the whole-body effective dose.

Effective dose (mSv)								
Beam diameter	Inside the beam diameter				Outside the beam diameter			
	n + γ	fraction	n only	fraction	n + γ	fraction	n only	fraction
Primary, 14 cm	47.6	46%	44.9	43%	56.1	54%	43.3	42%
Extended, 22 cm	71.0	69%	65.1	63%	32.5	31%	23.4	23%
Total	104							

Using ICRP 103 tissue weighting factor.

### 3.2 The beam profile and the ambient dose from the field edge

**Figure 3:**
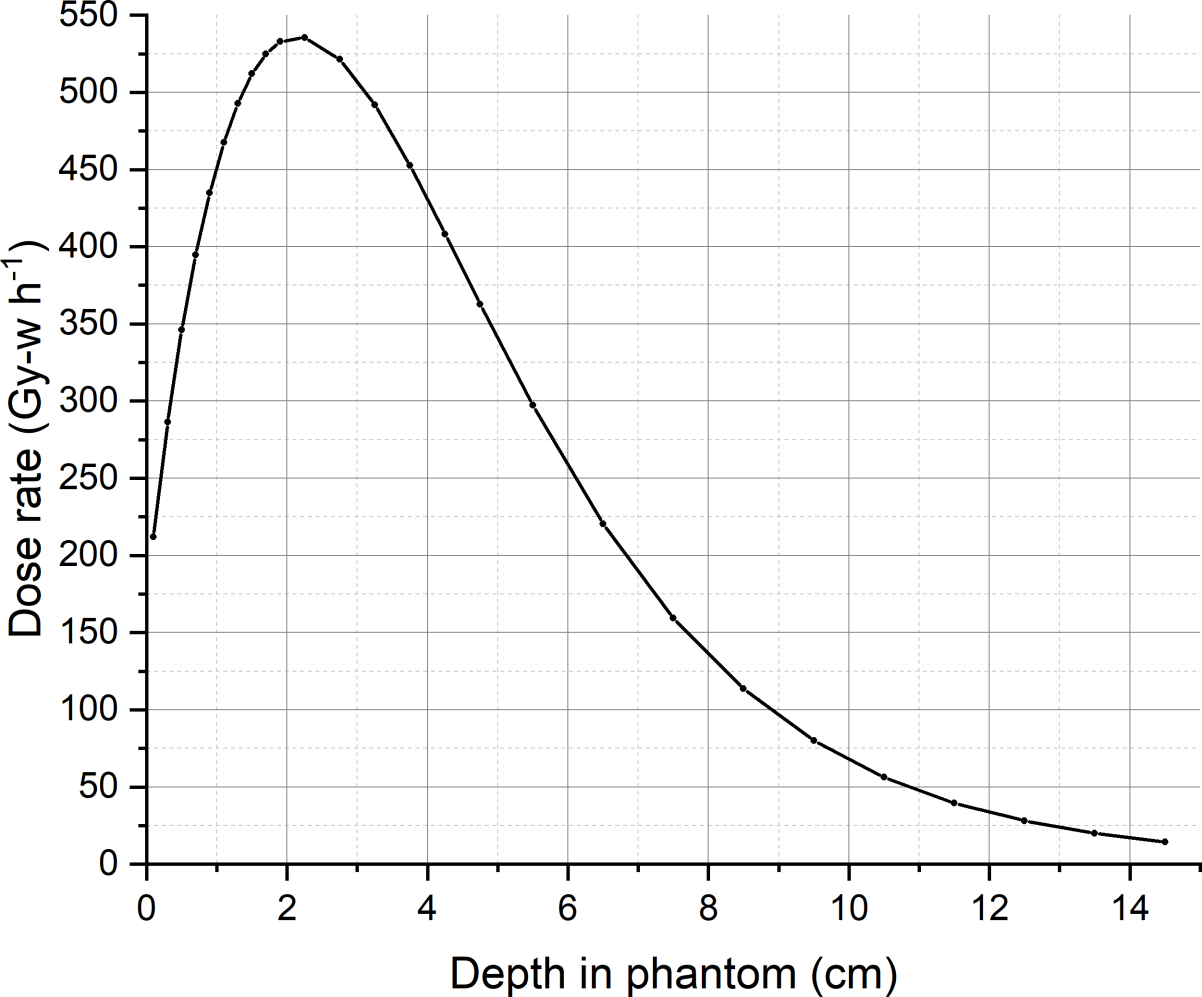
Tumor dose distribution in a water phantom placed at 2.75 cm from the neutron beam exit.

**Figure 4:**
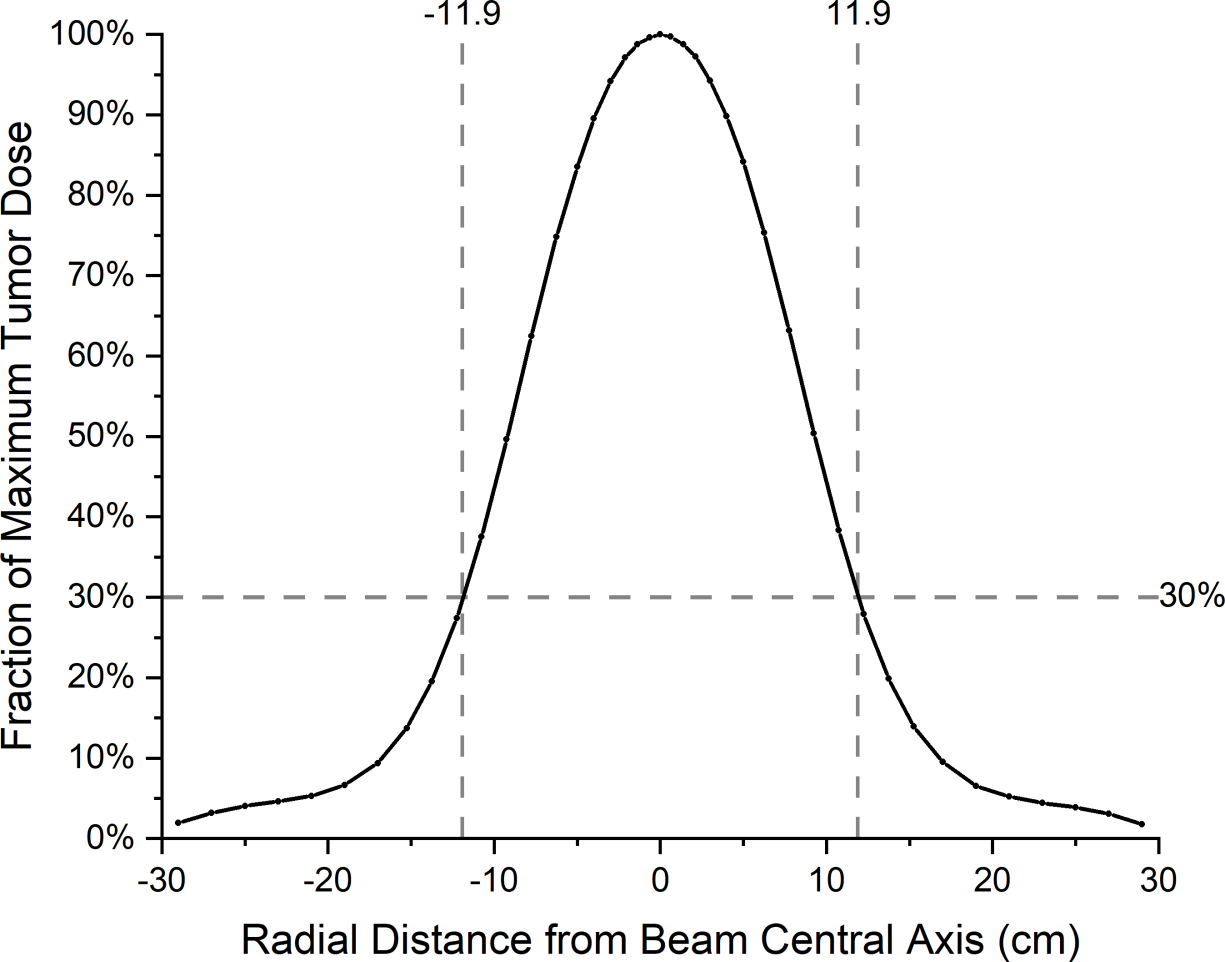
Radial profile of tumor dose at the depth of maximum of thermal neutron flux in the water phantom.

**Figure 5:**
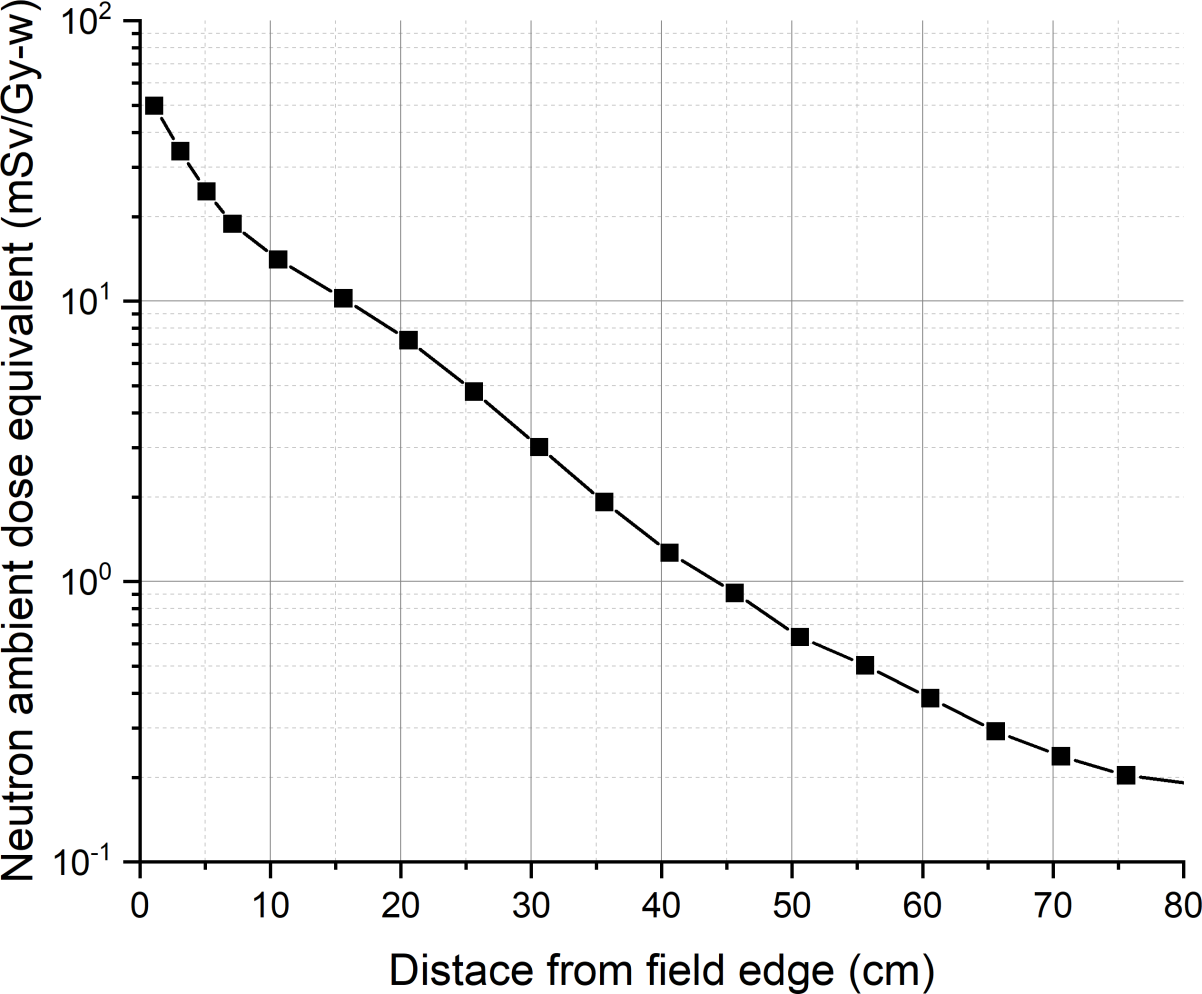
Neutron ambient dose equivalent from the field edge.

**Figure 6:**
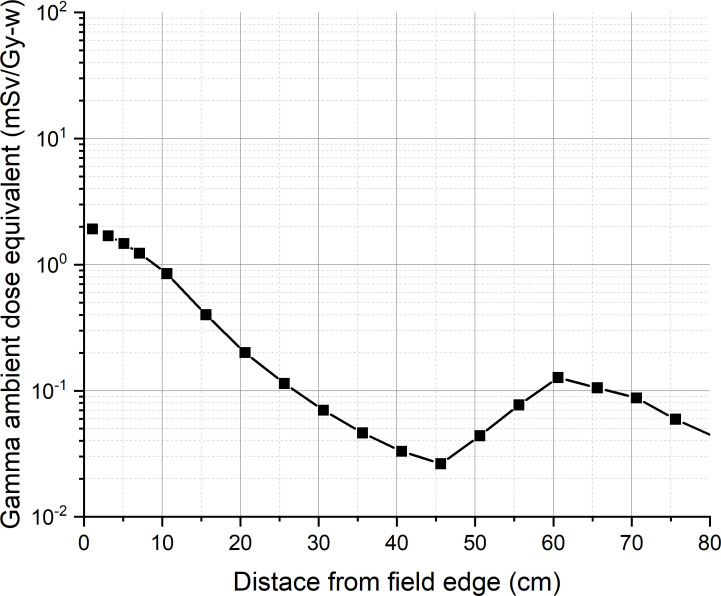
Gamma ray ambient dose equivalent from the field edge.

For calculating the ambient dose equivalent, the maximum tumor dose and beam edge parameter are required. A water phantom of 60 cm × 60 cm × 60 cm is placed at 2.75 cm from the designed epithermal neutron beam exit, so that the tumor dose maximum occurred at a depth of 5 cm from the beam exit. The maximum tumor dose occurred at a depth of 2.25 cm from the surface of the water phantom. **Figure 3** shows the tumor dose distribution in the water phantom. The maximum tumor dose rate is 535 Gy-w/h. **Figure 4** shows the radial profile of tumor dose at the maximum dose location. The field edge is found to be 11.9 cm. **Figure 5** shows neutron ambient dose equivalent from the field edge. The neutron ambient dose equivalent at 15 cm and 26 cm from the field edge is 11 mSv/Gy-w and 5 mSv/Gy-w, respectively. **Figure 6** shows the gamma ray ambient dose equivalent from the field edge. The gamma ray ambient dose equivalent is less than 1 mSv/Gy-w starting from 10 cm from the field edge.

Using the values of 3.2 for RBE of neutron and of 1 for RBE of gamma-ray, as commonly used in BNCT treatment planning, results in<1% difference in the maximum tumor dose rate. Because the tumor dose mainly comes from boron dose, the difference resulting from using different RBE of neutrons is minor. Because the RBE for neutron is related to the beam characteristics of each site, more commonly used values of RBE for neutron (3.2) and photon (1) are thought to be acceptable for use in the beam profile ambient dose equivalent calculation.

## 4 Discussion

The results of the out-of-field leakage of this study show that the gamma ray ambient dose equivalent is less than 1 mSv/Gy-w starting from 10 cm from the field edge. The neutron ambient dose equivalent is 11 mSv/Gy-w at 15 cm from the field edge and is <5 mSv/Gy-w starting from 26 cm from the field edge. Although the out-of-field neutron leakage of our present beam design is higher than that of the initial guideline proposed in Virtual Technical Meeting on Advances in Boron Neutron Capture Therapy held by IAEA on 27 to 31 July 2020, the value is less than 10% of the value at the center.

The results of whole-body dose calculation show that the effective dose of whole body is 104 mSv, and the RBE-weighted dose of organs in abdomen region is less than 40 mGy-w. As mentioned in ICRP 103 report, in the absorbed dose range up to 100 mGy (low or high linear energy transfer (LET)) no tissues are judged to express clinically relevant functional impairment. The results of the out-of-field leakage of our beam design are therefore considered acceptable.

In addition, the results show that 46% of the whole-body dose comes from the 14-cm-diameter real beam port and 69% of the dose comes from the virtually extended beam port region of 22 cm diameter. The neutron contribution to the total whole-body dose at the beam exit is 85%. For the dose from outside the 14-cm-diameter beam port, 77% is neutron dose, and half of them are from the ring region between radius of 7 cm and 11 cm. Further extending the neutron shielding at the peripheral of the beam exit may be useful in mitigating the out-of-field leakage.

## 5 Conclusions

Non-primary radiation should be considered during the BNCT neutron beam design for reducing patient risk such as cancer. Although the out-of-field leakage of the present beam design is higher than that in the initial guideline proposed in Virtual Technical Meeting on Advances in Boron Neutron Capture Therapy held by IAEA on 27 to 31 July 2020 (2), the whole-body dose, however, is reasonably low. The whole-body dose calculation along with the analysis of dose contribution from the non-primary beam is useful in beam design consideration. The whole-body dose calculation together with the beam profile analysis can also be helpful in reaching an acceptable recommendation for the out-of-field leakage for BNCT neutron beam, a job wished to be accomplished in the near future as pointed out in the 2023 IAEA’s report on Advances in Boron Neutron Capture Therapy (3).

## Data availability statement

The datasets presented in this article are not readily available because the dataset is property of the company, will need special permission from the top level. Requests to access the datasets should be directed to Yvonne.Hsueh @heron-neutron.com.

## Author contributions

Z-FY: Conceptualization, Data curation, Formal analysis, Investigation, Methodology, Resources, Visualization, Writing – original draft. C-KH: Data curation, Formal analysis, Investigation, Methodology, Writing – review & editing, Visualization. Y-WL: Conceptualization, Funding acquisition, Project administration, Resources, Supervision, Visualization, Writing – review & editing.
